# OP73 Navigating The Advanced Therapy Medicinal Products (ATMPs) Conundrum: Insights From ATMPs Withdrawn In The European Market

**DOI:** 10.1017/S0266462324001326

**Published:** 2025-01-07

**Authors:** Katherine Leong, Richard Macaulay

## Abstract

**Introduction:**

Advanced therapy medicinal product (ATMP) is a classification used by the European Medicines Agency (EMA) for cell-, gene-, and tissue-engineered therapies. Since the first ATMP received market authorization in 2009, a substantial proportion of these highly innovative therapies have been withdrawn from the European market. This research investigates the key reasons underlying these withdrawals.

**Methods:**

ATMPs with withdrawn EMA marketing authorizations were identified from the EMA website. A targeted review of relevant company press releases was undertaken and key information extracted. Health technology assessment (HTA) outcomes in France, Germany, and England were extracted from their respective websites (1 Sep 2023).

**Results:**

Thirty-two ATMPs have received EMA marketing authorizations, with 22 percent (7/32) withdrawn. One of these seven withdrawals was driven by unfavorable clinical results versus six out of seven due to commercial reasons. Of the six withdrawals driven by commercial reasons, four were associated with negative HTA and/or reimbursement issues (Chondro-Celect, Glybera, and Zalmoxis’s Service Médical Rendu (SMR, actual medical benefit) insufficient, driven by data-related issues in France; Glybera’s challenge to obtain reimbursement by insurance funds with only one sale reported, and Zynteglo’s price agreement not reached in Germany). One of the six withdrawals (Chondro-Celect) was also associated with hospital exemption (continued production without marketing authorization). One of six (Provenge) was also associated with chemistry-, manufacturing-, and controls (CMC)-related issues.

**Conclusions:**

The ATMP landscape has rapidly evolved in the past decade. While ATMPs offer the promise of long-term benefits, the unique manufacturing, clinical, and especially the reimbursement challenges they face can lead to commercial failure. To ensure ATMP patient access and commercial success, manufacturers should engage early with payers, understand potential reimbursement challenges, and proactively plan to mitigate these.

